# Statistics of Medical Colleges

**Published:** 1859-08

**Authors:** 


*Statistics of Medical Colleges.*—In comparing the number of students in attendance at the Medical Colleges for the past season with that of the year before, we see quite a decided increase. All the institutions have not yet been heard from, but we have quite a full report in the last number of the "Southern Medical and Surgical Journal;" and last year's report is to be found in our issue for July, 1858. Last year, eleven colleges were reported with a total number of 2,437 students. The number of students in attendance at the same institutions this year, has been 2,713, making an increase of 276 students. The reports of graduates is more complete for both years. Nineteen colleges last year had 1,111 graduates, while the same institutions this year had 1,185, making an increase of 74. We are unable to present full reports of either year; but the figures which we have given, show a marked increase in the number of students in the country.

